# A Comparison of RML Prion Inactivation Efficiency by Heterogeneous and Homogeneous Photocatalysis

**DOI:** 10.3390/pathogens13050420

**Published:** 2024-05-16

**Authors:** Ioannis Paspaltsis, Eirini Kanata, Sotirios Sotiriadis, Susana Silva Correia, Matthias Schmitz, Inga Zerr, Dimitra Dafou, Konstantinos Xanthopoulos, Theodoros Sklaviadis

**Affiliations:** 1 Laboratory of Pharmacology, Department of Pharmacy, School of Health Sciences, Aristotle University of Thessaloniki, 54124 Thessaloniki, Greece; ipaspalt@pharm.auth.gr (I.P.); ekanata@pharm.auth.gr (E.K.); sotiriadis@bio.auth.gr (S.S.); 2 Department of Neurology, University Medicine Goettingen, German Center for Neurodegenerative Diseases (DZNE), 37075 Göttingen, Germany; 3 Department of Genetics, Development and Molecular Biology, School of Biology, Aristotle University of Thessaloniki, 54124 Thessaloniki, Greece; 4 Centre for Research and Technology Hellas, Institute of Applied Biosciences, 57001 Thermi, Greece

**Keywords:** RML prion, homogeneous photocatalysis, heterogeneous photocatalysis, photo-Fenton reagent, TiO_2_ photocatalysis, prion inactivation, prion decontamination

## Abstract

Prions are proteinaceous pathogens responsible for a variety of devastating diseases in mammals, including scrapie in sheep and goats, chronic wasting disease in cervids, and Creutzfeldt–Jakob disease (CJD) in humans. They are characterized by their exceptional persistence to common inactivation procedures. This applies to all possible sources of prion contamination as prions may be present in the tissues and biological fluids of infected individuals. Hence, efficient prion inactivation procedures are still being sought to minimize the risk of intra- or inter-species transmission. In the past, photocatalytic treatment has been proven to be capable of efficiently oxidizing and inactivating prions. In the present study, the efficacy of homogeneous photo-Fenton-based photocatalysis as well as heterogeneous photocatalysis with TiO_2_ in reducing RML mouse scrapie infectivity was evaluated. Prion inactivation was assessed by means of a bioassay, and the results were confirmed by in vitro experiments. While the prion infectivity of the RML mouse scrapie was reduced after treatment with the photo-Fenton reagent, the heterogeneous photocatalytic treatment of the same prion strain completely eliminated prion infectivity.

## 1. Introduction

Prions are the causative agents of several fatal transmissible neurodegenerative disorders, known as Transmissible Spongiform Encephalopathies (TSEs) [1,2,3]. Pathogenesis is characterized by the accumulation of an abnormally folded isoform of the prion protein (PrP^Sc^) in the CNS, which is generated by the misfolding of the physiological host-encoded cellular isoform (PrP^C^) [4]. The infection and further propagation mechanism of prion diseases are associated with the capacity of PrP^Sc^ to interact with PrP^C^ and induce its structural modification to an emerging misfolded PrP^Sc^ molecule [5]. Accordingly, newly formed PrP^Sc^ molecules can direct the conversion of the normal to the pathological isoform, further propagating the disease. The structural conversion of PrP^C^ to PrP^Sc^ is associated with differences in physicochemical and biochemical properties, including solubility, resistance to proteinase K, and propensity to form fibrils [6].

The infectious agent can be transmitted in several ways, including the ingestion of contaminated food [7], administration of prion-contaminated human growth hormones, blood transfusion, and reuse of contaminated neurosurgical tools [8]. In addition, medical liquid waste, especially waste originating from pathology laboratories, may include prions and thus represents a source of prion infectivity shedding. Although prion diseases are extremely rare, [9], prion contagion poses a public health concern, particularly for individuals undergoing surgery, healthcare providers, and personnel working in laboratories.

The prion pathogen’s unique features dictate different and considerably harsher procedures compared to those applied to other pathogenic agents for its inactivation. Prions are remarkably stable protein structures, which are devoid of nucleic acid, and for their inactivation, various methods have been suggested, including autoclaving in higher temperature/pressure (134 °C for 18 min) or treatment with (i) sodium hydroxide (NaOH); (ii) alkaline detergent; (iii) phenolic disinfectant [10]; (iv) sodium hypochlorite (NaOCl) [11]; (v) 3% sodium dodecyl sulfate (SDS); (vi) 7 M guanidine hydrochloride/3 M guanidine thiocyanate/3 M trichloroacetic acid; (vii) 60% and 80% formic acid; (viii) 50% phenol [12]; (ix) enzyme detergent (with hydrogen peroxide gas plasma sterilization) and vaporized hydrogen peroxide [7,13,14]; (x) hydrogen peroxide gas plasma (Sterrad NX); and (xi) sodium metaperiodate, quaternary ammonium compound, and peracetic acid [15].

In the context of sustainable development and alternative technologies, photocatalytic oxidation has emerged as a viable solution for the inactivation of prions. Photocatalytic oxidation, an advanced oxidation process (AOP), is based on the production and non-selective effect/action of reactive transient substances or reactive oxygen species (ROS), including hydroperoxyl (HO_2_·), superoxide (O_2_^−^), and hydroxyl (HO˙) radicals [16,17], which react with organic molecules, eventually resulting in H_2_O, mineral acids, and CO_2_ (mineralization) [18]. Photocatalysis is divided into two main categories depending on the photocatalyst’s phase: homogeneous and heterogeneous. In homogeneous photocatalysis, inactivation is achieved by the irradiation of the catalyst, such as the Fenton reagent (an H_2_O_2_ and Fe^2+^ mixture), which remains soluble in acidic conditions. This leads to the generation of O_2_^−^ and HO˙. Hydroxyl radicals are typically produced through reactions involving oxidizing agents like H_2_O_2_ [19]. In heterogeneous photocatalysis, under irradiation with a wavelength equal to or greater than the energy gap of the photocatalyst (such as TiO_2_), the electrons from the valence band are stimulated to the conduction band, and oxidative holes (h+) are created in the valence band. The photogenerated released electrons bind to oxygen on the photocatalyst’s surface, reducing it to superoxide anion radicals (O_2_^−^). Simultaneously, due to photogenerated holes, surface HO^−^ groups are oxidized and converted into hydroxyl radicals (HO·) [20].

Our research group has already performed several studies in order to investigate the potential of photocatalysis as an effective alternative method to inactivate prions with encouraging results. In this context, TiO_2_ used as a photocatalyst, has been proven to significantly reduce PrP^Sc^ infectivity by using the 263K experimental scrapie model in hamsters. UV-A illumination of 1% *w*/*v* 263K scrapie homogenate in the presence of 4 g L^−1^ TiO_2_ for 12 h resulted in a 25% survival of hamsters in an ensuing bioassay [21]. Moreover, treatment with the photo-Fenton reagent eliminated or significantly reduced PrP^Sc^ 263K hamster scrapie infectivity attached to stainless steel or titanium metal wires, respectively, which were stereotactically implanted in their brains. Notably, such metals are commonly used in the manufacture of surgical instruments. To achieve these results, artificially prion-contaminated wires were illuminated with UV-A in the presence of 224 mg L^−1^ FeCl_3_ and 500 mg L^−1^ H_2_O_2_ for 8 h [22]. Meanwhile, the non-specific protein degradation occurring during the photocatalytic treatment with the photo-Fenton was confirmed in experiments using recombinant PrP proteins, as well as whole protein extracts originating from diseased species in different natural or experimental TSEs as a proteinaceous substrate [23].

Here, we report the results on both the homogeneous (FeCl_3_) and heterogeneous (TiO_2_) photocatalytic degradation of PrP and RML infectivity in aqueous suspensions (brain homogenates) to simulate prion as a contaminant in medical liquid wastewater effluents and evaluate the applicability of photocatalytic prion degradation in liquid waste originating from pathological and biochemical laboratories.

## 2. Materials and Methods

### 2.1. Preparation of Infectious Material

Mouse RML scrapie prions were propagated in C57BL/6 mice. The brains from animals at the terminal stage of the disease were collected and homogenized in PBS (phosphate-buffered saline), with a pH of 7.4, 0.5% *w*/*v* sodium deoxycholate, 0.5% *w*/*v* Igepal, and 5 mM phenylmethanesulphonylfluoride to obtain a 10% *w/v* homogenate. Homogenization was performed in 2.0-mL lysing matrix tubes (MP Biomedicals, 115076200-CF, Eschwege, Germany) loaded with 1.0-mm zirconia–silica beads (BioSpec, 11079110Z, Bartlesville, OK, USA) using the Ribolyzer apparatus (Hybaid, Heidelberg, Germany) for 3 × 40 s pulses with a setting of 4. The lysates were centrifuged for 5 min at 1500× *g* to pellet the insoluble matter. The supernatant was aliquoted and stored at −80 °C for the ensuing experiments.

### 2.2. Photocatalyses

Heterogeneous photocatalytic experiments were conducted with TiO_2_ P25 (Evonik, Weston, MI, USA; 70% anatase–30% rutile, BET 55 ± 15 m^2^ g^−1^, and crystallite size 15 nm) suspended in PBS. Then, a 1% *w*/*v* TiO_2_ stock suspension in H_2_O was diluted to a final content of 0.5 g L^−1^. The photo-Fenton reagent used for homogeneous photocatalytic experiments consisted of FeCl_3_ (Chem-Lab, Zedelgem, Belgium) and H_2_O_2_ under UV-A illumination. A 7 g L^−1^ stock FeCl_3_ solution in H_2_O at a pH of 3 was used. To ensure FeCl_3_ solubility, the pH of the diluent in all homogeneous photocatalytic experiments was set to 3.

All the experiments were performed in disposable standard 6 well plates for cell culture, and the final volume of the reaction was 10 mL. The reaction mixture containing the respective catalyst, H_2_O_2_, RML brain homogenate, and diluent (H_2_O with a pH of 3 or PBS) was maintained each time in a state of suspension through constant stirring at 800 rpm. A bench-scale photocatalytic reactor was used, and reaction plates were placed 10 cm away from the irradiation source. Illumination was provided by five parallel UV-A lamps (TLD 8 W/08, Phillips, emitting light with a spectral peak centered on 365 nm, 30 cm long, and connected to a voltage stabilizer). The average light intensity measured with a luminometer was 4.5 mW cm^−2^. H_2_O_2_ content in the reactions was monitored periodically, i.e., every 2 h, with colorimetric peroxide test strips (MQuant, Merck, Darmstadt, Germany) and adjusted to the given initial concentration (1000 mg L^−1^).

In all the photocatalytic experiments, 10% *w*/*v* RML mouse brain homogenate was diluted 100-fold to yield a final concentration of 0.1% *w*/*v* in the reaction (10 mg of brain equivalent per 10 mL). For sampling, the indicated volume of the reaction was withdrawn. Proteins were concentrated by methanol precipitation. After the addition of 100% methanol (9× volumes), the samples were incubated overnight at −80 °C. Furthermore, the samples were centrifuged at 10,000× *g* for 15 min, and the pellet was diluted in 2 × O’Farrell sample buffer for subsequent electrophoresis [24].

### 2.3. Protein Electrophoresis and Immunoblotting 

Photocatalytic PrP degradation was monitored by western blotting as previously described [21]. Briefly, proteins from treated or untreated control samples were resolved in 12% polyacrylamide gels by SDS-PAGE and subsequently transferred onto polyvinylidene fluoride (PVDF) membranes. As the primary antibody for immunoblotting, a mouse monoclonal anti-PrP antibody 6H4 (epitope DYEDRYYRE corresponding to amino acids 144–152 of human PrP, while also recognizing the DWEDRYYRE sequence corresponding to amino acids 143–151 in the murine PrP) diluted in blocking buffer 1:5000 *v*/*v* was used, followed by a HRP-conjugated goat anti-mouse (Sigma, A0168, Saint Louis, MI, USA) secondary antibody, diluted to 1:50,000 *v*/*v*. The SignalFire ECL reagent (Cell Signaling Technology, Danvers, MA, USA), which enables protein detection at the picogram level, was used for the visualization of the immunoblot, according to the manufacturer’s instructions. Since samples were not treated with proteinase K, the signal obtained after the described immunostaining corresponds to the initial or remaining total PrP (PrP^C^ + PrP^Sc^) in each sample. Densitometric analysis was performed with the Image J software (https://imagej.net/ij/, accessed on 2 April 2024) [25].

### 2.4. Bioassay 

**Scheme 1:**
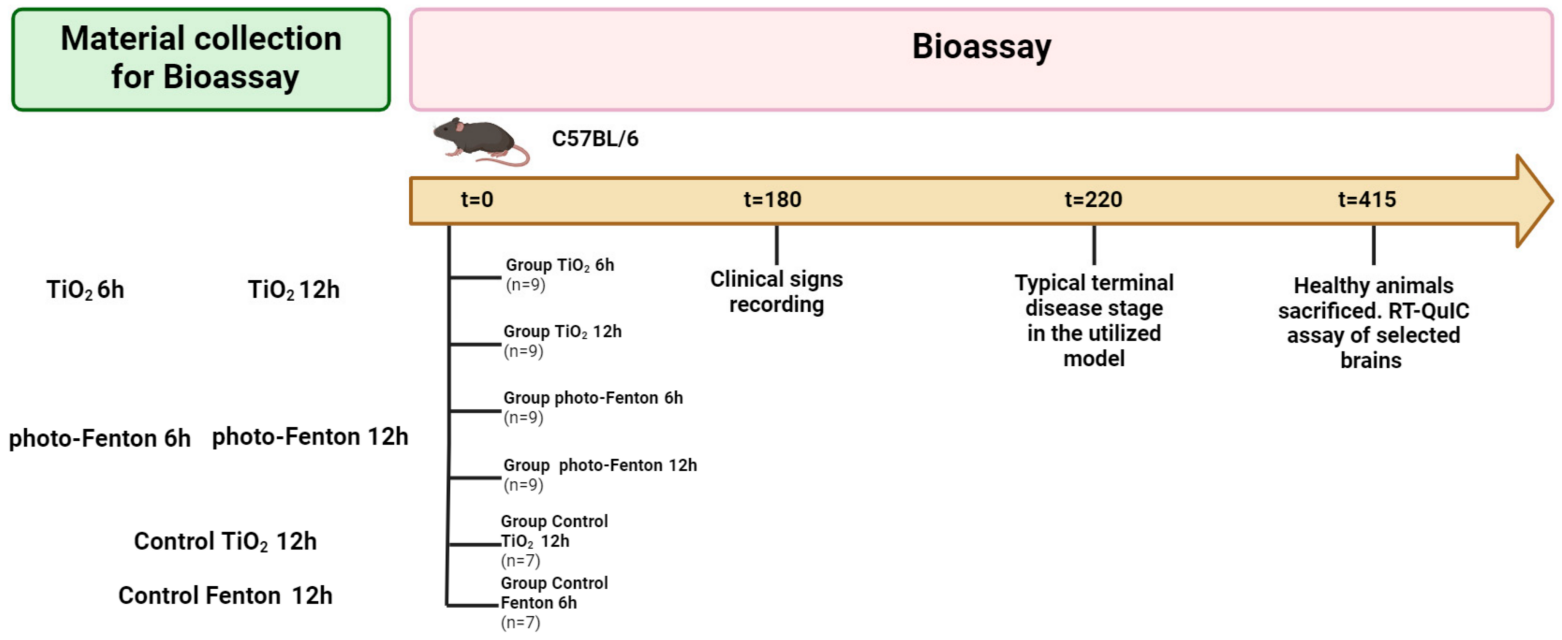
Detailed schedule of the bioassay. The mice were divided into six groups of 7−9 individuals each depending on the type and duration of the treatment. The control groups (control TiO_2_ and control photo-Fenton) received the materials contained in heterogeneous and homogeneous photocatalytic reactions under stirring for the maximal treatment duration (12 h) in dark conditions. The observation for the occurrence of typical scrapie symptoms started at 120 dpi (days post inoculation) and intensified after 180 dpi as RML mice typically reach the terminal stage at about 220 dpi. The experiment stopped at 415 dpi, and animals that were still healthy were sacrificed.

The effect of photocatalytic treatment on prion infectivity was assessed by a bioassay. The animal experimentation was conducted in compliance with the guidelines and regulations stipulated by the local ethics committee (Veterinary Services of the District of Central Macedonia, approval code: 542188(2288) and approval date: 7 October 2020). Female C57BL/6 mice that were 6–8 weeks old were divided into six groups consisting of 7–9 individuals each. The timeline of the assay is summarized in Scheme 1. In each animal, 100 μL of homogeneously (56 mg L^−1^ FeCl_3,_ 1000 mg L^−1^ H_2_O_2_, pH 3) or heterogeneously (0.5% *w*/*v* TiO_2_, 1000 mg L^−1^ H_2_O_2_, pH 7.4) treated 0.1 % *w*/*v* mouse RML prion homogenate was intraperitoneally injected. This volume initially contained the infectivity of 0.1 mg of brain tissue, corresponding to an infectivity of approximately 10^3.5^xLD_50_. Photocatalytic treatment was performed for either 6 h or 12 h, while two animal groups served as controls. Animals of those groups were also intraperitoneally injected with material originating from control reactions, which contained all the ingredients as above. Control reactions were performed for 12 h in the absence of UV-A illumination.

Following inoculation, the mice were constantly monitored for the appearance of symptoms associated with the RML strain. To quantify disease progression, a symptom scale was used (summarized in Supplementary Figure S1), and a clinical score was assigned to each mouse. Mice were sacrificed at the terminal stage, which was defined as a clinical score of 4 or higher, and survival curves showing the percentage of mice remaining alive vs. the days post inoculation were prepared. The survival curves were compared pairwise vs. the corresponding control (i.e., groups receiving material photocatalytically treated for 6 h vs. control and groups receiving photocatalytically treated material for 12 h vs. control) with the log-rank (Mantel–Cox) test using GraphPad Prism (version 9.1.2) software.

The mice were observed for the appearance of typical RML symptoms, as detailed in Supplementary Table S1. Clinical scores of 1 were not associated with RML infection but rather with other factors such as aging, whereas mice reaching a clinical score of 3 and above were nearing the terminal stage (mice were usually sacrificed at a clinical stage of 4).

### 2.5. RT-QuIC Assay

For the RT-QuIC assay, a standard protocol was used, as previously described [26,27]. Briefly, hamster–sheep recPrP was used as the substrate to amplify and detect any PrP^Sc^ present in 10% *w*/*v* brain homogenates from the mice in the bioassay. Homogenates were diluted to 10^−6^ in RT-QuIC buffer (165 μL of ddH_2_O, 100 μL of 5X PBS buffer, pH 6.9, 22 μL of NaCl 5 M, and 1 μL of EDTA 0.5 M). Each reaction consisted of 15 µL of diluted brain homogenate, 57 µL of RT-QuIC Buffer, 1 µL of 1 mM Thioflavin-T, and 27 µL of recHaShPrP^C^ (0.37 mg mL^−1^) to a final volume of 100 µL. The reactions were prepared in a black 96-well, Nunc optical-bottomed plate (Nalgene, Conway, NH, USA). The plates were sealed and incubated in a BMG OPTIMA FLUOStar plate reader at 42 °C for 80 h with intermittent shaking cycles consisting of one minute of double orbital shaking at the highest speed (600 rpm), followed by 1 min of rest. Fibril formation kinetics were determined by measuring the Thioflavin-T fluorescent signal (450 nm excitation and 480 nm emission) every 30 min. Each sample was analyzed in triplicate and was considered to be positive when at least 2 of the 3 replicates were positive.

## 3. Results

**Figure 1:**
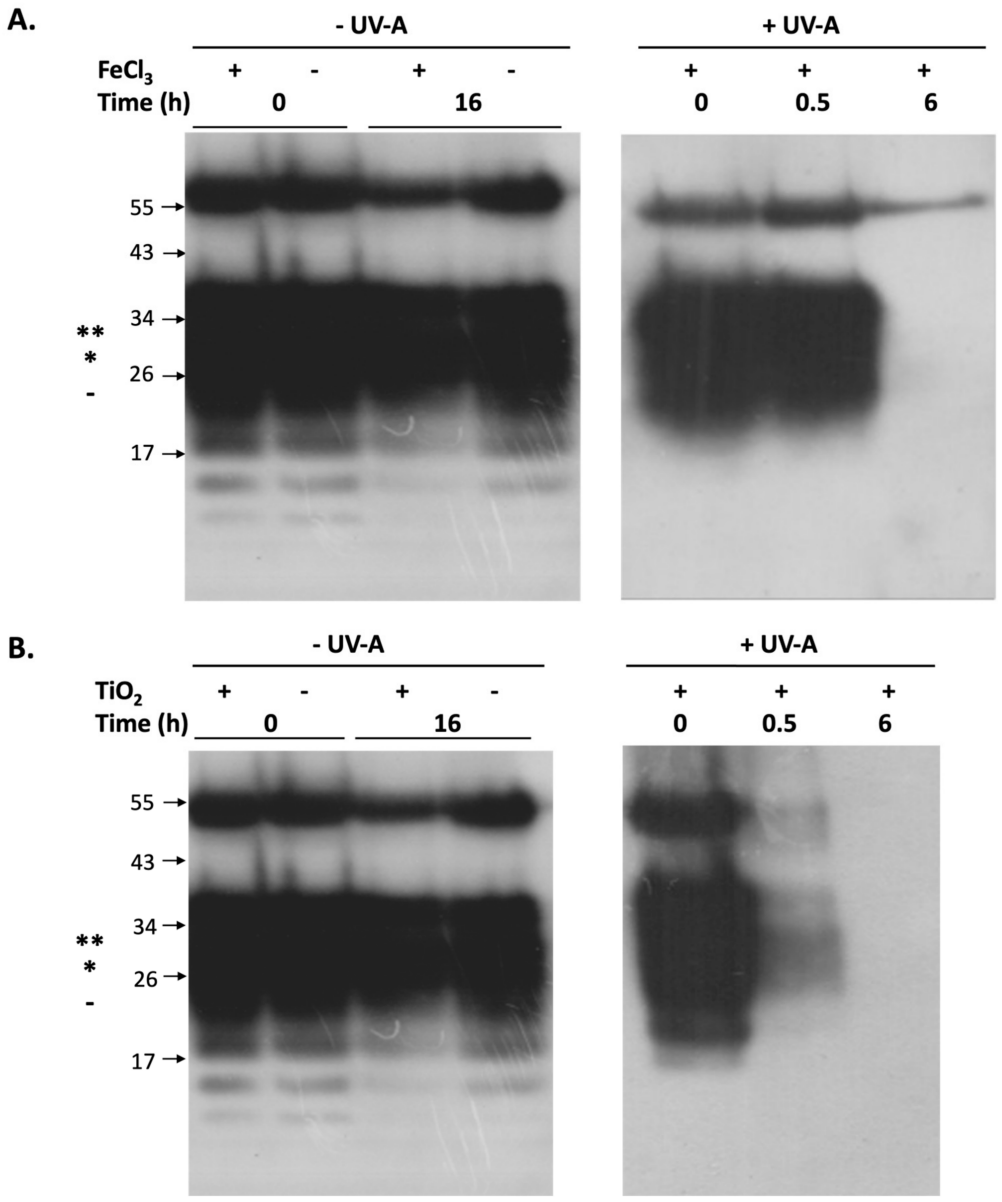
Western blot detection of total PrP (PrP^C^ + PrP^Sc^) after (**A**) homogeneous (56 ppm FeCl_3_, 1000 ppm H_2_O_2_, and UV-A) or (**B**) heterogeneous (0.5% *w*/*v* TiO_2_, 1000 ppm H_2_O_2_, and UV-A) photocatalytic treatments compared to control reactions in the absence of UV-A. The PrP protein signal in the control samples and at the time point 0 corresponds to the 0.6-mg brain tissue equivalent. For the 0.5 and 6 h photocatalytic treatments, 1 mg and 2 mg brain equivalents were loaded, respectively. Primary antibody: 6H4 diluted 1:5000. Secondary antibody: goat anti-mouse-HRP diluted 1:50,000. Arrows indicate the positions of the pre-stained relative molecular weight standards (kDa). The diglycosylated (**), monoglycosylated (*), and unglycosylated PrP bands are indicated.

Initial in vitro experiments were carried out to assess the ability of either homogeneous or heterogeneous photocatalysis to oxidize, nonspecifically, the PrP protein, among other brain proteins (Figure 1). After 6 h of photocatalytic treatment, both methods significantly decreased the detected PrP signal in immunoblots compared to control reactions in the presence of the catalyst but without UV-A illumination. 

**Figure 2:**
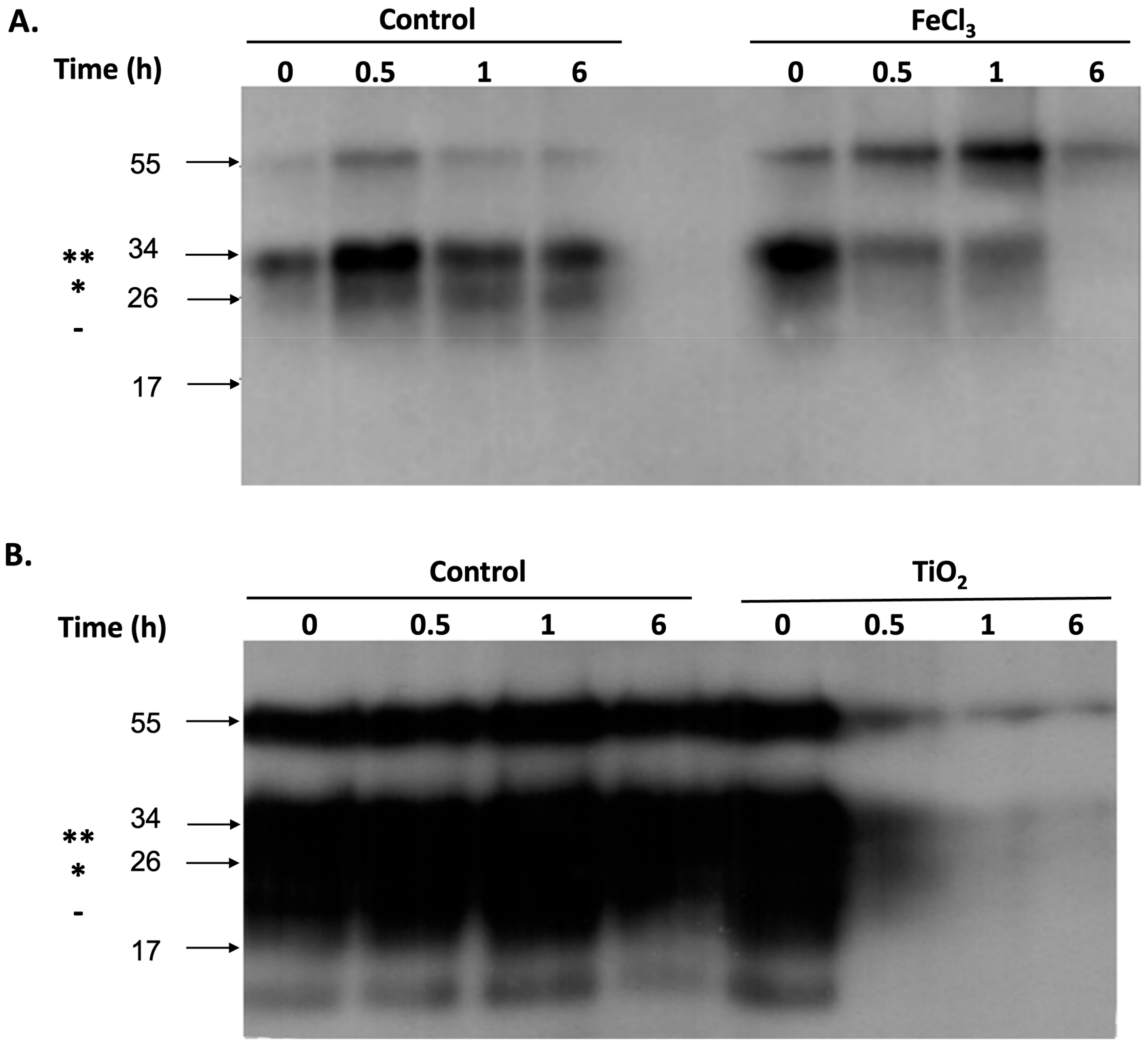
Immunoblot of the inocula used for the bioassay. Total PrP protein (PrP^C^ + PrP^Sc^) signal after (**A**) homogeneous photocatalytic treatment with 56 ppm FeCl_3_, 1000 ppm H_2_O_2_, and UV-A and (**B**) heterogeneous photocatalytic treatment with 0.5% *w*/*v* TiO_2_, 1000 ppm H_2_O_2_, and UV-A, which, in each panel, are compared to control reactions in the absence of UV-A. The PrP protein signal in the control lanes as well as the time point 0 lane corresponds to the 0.6-mg brain tissue equivalent. For 0.5, 1, and 6 h of photocatalytic treatment, materials equivalent to 1.2 mg, 1.8 mg, and 2.4 mg of brain tissue were loaded, respectively. Primary antibody: 6H4 diluted 1:5000. Secondary antibody: goat anti-mouse-HRP diluted 1:50,000. Arrows indicate the positions of the relative molecular weight standards (kDa). The diglycosylated (**), monoglycosylated (*), and unglycosylated PrP bands are indicated.

PrP protein has two potential N-glycosylation sites [6]. Thus, the three different immuno-positive bands identified in western blots correspond to the di-, mono-, and un-glycosylated forms of the protein [28]. We also detected an immuno-positive band at approximately 55 kDa, which could represent PrP dimers. Densitometric analysis of the bands in the blot depicted in Figure 2A indicates that the ratio of the signal corresponding to the dimer vs. the diglycosylated form rises for the first 1 h of photocatalytic treatment (0 h: 0.33; 0.5 h: 0.85; and 1 h: 1.07) and is then significantly reduced at 6 h (the ratio cannot be calculated due to the oxidation of the diglycosylated form). Dimer formation is expected to hamper the access of ROS to the protein core, enhancing the stability of the dimeric form of the protein compared to the monomeric form.

To validate the efficacy of both photocatalytic methods in vivo, we utilized the conditions determined in Figure 1 to prepare the photocatlytically treated material to be used as inocula in the bioassay. Inocula treatment was performed as before, and material treated for 6 h (where reduced PrP levels are detected by WB, Figure 2) or 12 h was used for the bioassay. Control reactions performed in the presence of catalysts but in the absence of UV-A illumination for 12 h were used in the bioassay. During treatment, the material was sampled at different time points and used in western blot analysis for the immunodetection of PrP (Figure 2). Similarly to the previous experiments, no PrP signal was detected following a 6-h homogeneous or heterogeneous photocatalytic treatment.

**Figure 3:**
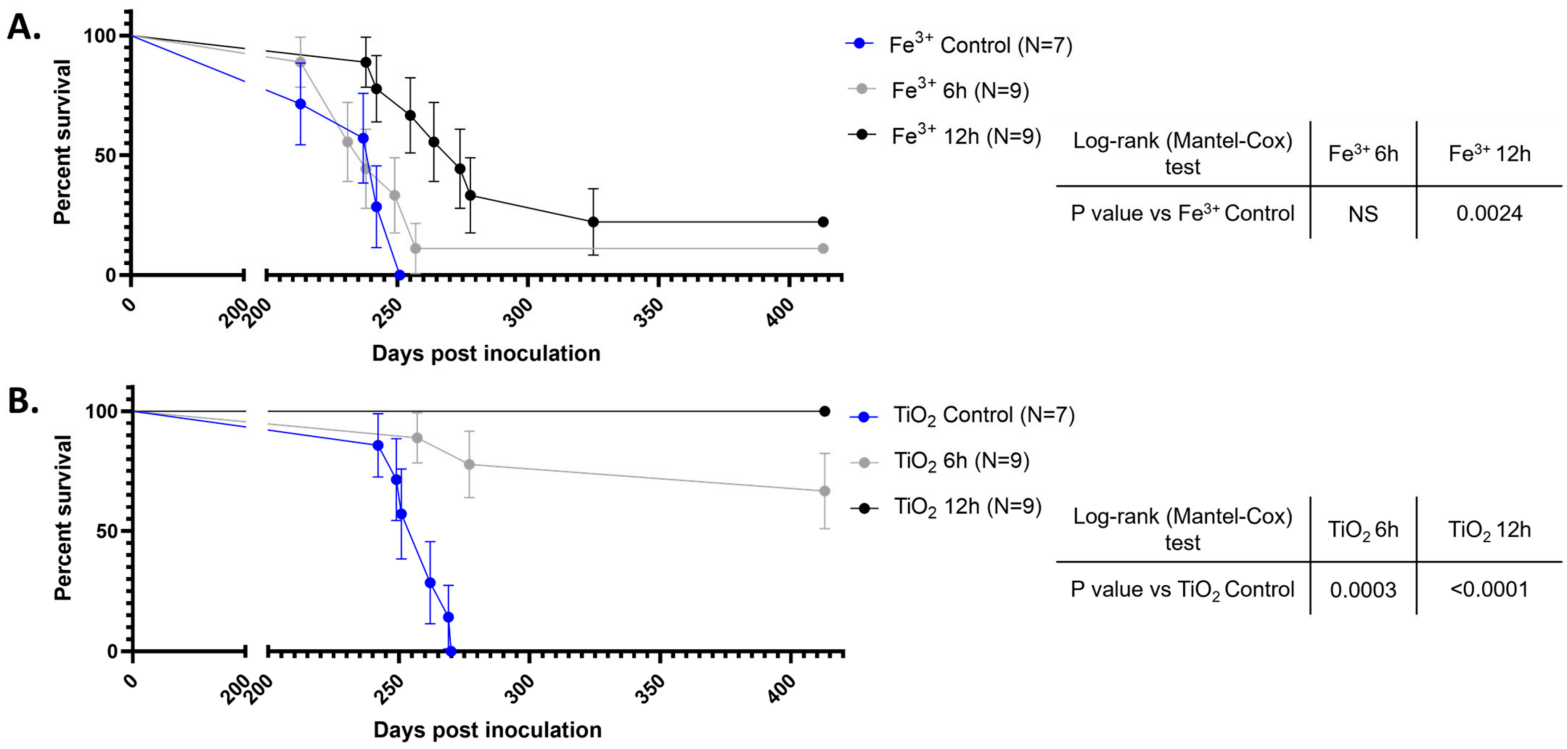
Survival curves of the groups of mice receiving photocatalytically treated inocula. (**A**) Mice receiving inocula after 6 h (grey line, N = 9) or 12 h (black line, N = 9) of homogeneous photocatalytic treatment. (**B**) Mice receiving inocula after 6 h (grey line, N = 9) or 12 h (black line, N = 9) of heterogeneous photocatalytic treatment. In both cases, control groups (blue lines) consisted of N = 7 animals receiving inocula prepared after 12 h treatment in the presence of the catalyst (FeCl_3_ or TiO_2_ for homogeneous and heterogeneous reactions, respectively) but without UV-A illumination. The survival curves for each group were compared pairwise with the corresponding control group using the log-rank (Mantel–Cox) test, and the p-value for each test is given in the tables (NS: not statistically significant).

To further assess the pathogenicity of the treated material, 0.1 mg of the brain equivalent of the treated or control material was injected intraperitoneally into mice, as summarized in Scheme 1. This tissue amount was initially contained in 100 μL of each photocatalytic or control reaction setup. The mice were observed for the appearance of typical RML symptoms, as detailed in Supplementary Table S1. A clinical score of 1 was not associated with RML infection but rather with other factors such as aging, whereas mice reaching a clinical score of 3 and above were nearing the terminal stage (mice were usually sacrificed at clinical stage 4). The average clinical score progression for each group is depicted in Supplementary Figure S1, while the survival curves for each group in the bioassay are summarized in Figure 3. As expected, all animals in the control groups, wherein the photocatalytic treatment was performed in the absence of UV-A illumination, displayed typical RML symptoms and succumbed to disease. Strikingly, a 100% survival rate was observed for the group of animals inoculated with material that was photocatalytically treated for 12 h in the presence of TiO_2_ 415 days after inoculation, with all animals of this group being symptom-free, suggesting RML prion infectivity elimination. A modest reduction in RML infectivity was observed in animals inoculated with material photocatalytically treated for 6 h in the presence of TiO_2_, with the survival rate exceeding 65%. On the other hand, both groups receiving material photocatalytically treated for either 6 or 12 h in the presence of FeCl_3_ displayed only modest increases in the survival rates compared to controls, which did not reach statistical significance. These data indicate that TiO_2_-based photocatalytic oxidation efficiently reduces RML prion infectivity.

**Figure 4:**
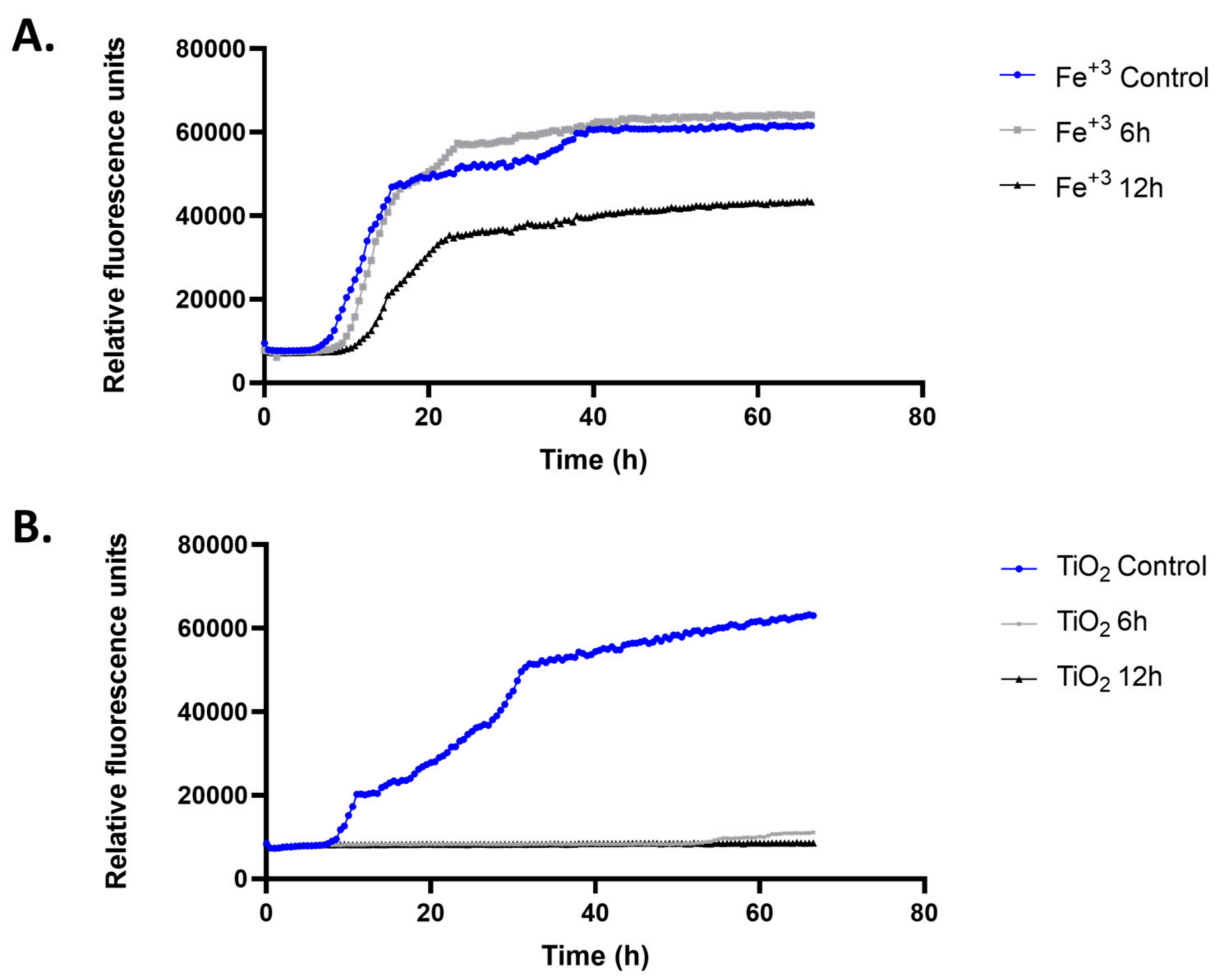
TiO_2_-treated brain samples showed no seeding activity in the RT-QuIC assay. Brain homogenates from 2 or 3 animals per group were used as the template in RT-QuIC reactions. The graphs illustrate the fluorescence intensity resulting from thioflavin binding to PrP aggregates plotted against time following homogeneous (**A**) or heterogeneous (**B**) photocatalytic treatments. The values represent the mean fluorescence intensity measured for each mouse tested in the respective group. An increase in fluorescence over time was observed in homogenates containing PrP^Sc^, as seen in the homogeneous photocatalysis graph. Conversely, the absence of PrP^Sc^, achieved through the complete inactivation of the contaminant via photocatalysis treatment, did not yield an increase in fluorescence over time, particularly evident in the case of heterogeneous photocatalysis (6 and 12 h).

To further consolidate our findings, we performed RT-QuIC assays in brain homogenates originating from the mice in the bioassay. For each group in the bioassay, brain homogenates (n = 3 or 2) were tested. The analysis was performed on randomly selected mice, either to ascertain that mice succumbing to disease accumulated abnormally folded PrP or to show that abnormally folded PrP was not present in mice not displaying RML symptomatology. Thus, for mice receiving material photocatalytically treated in the presence of TiO_2_, all the samples originated from mice that did not succumb to disease, whereas for mice receiving material treated in the presence of FeCl_3_, both terminally ill and mice that did not succumb to the disease were included. Consistent with the bioassay data, no seeding activity was observed in reactions seeded with brain homogenates from mice that were challenged with material photocatalytically inactivated for either 6 or 12 h in the presence of TiO_2_. This observation indicates the absence of abnormally folded PrP in the brain homogenate (Figure 4B). Similarly, in samples from mice receiving the FeCl_3-_treated material, robust seeding activity, similar to controls, was observed in samples from terminally ill mice, while samples from asymptomatic mice failed to promote PrP aggregation (Figure 4A and Table 1). These data dismiss the possibility that experimental animals lacking symptoms were asymptomatic carriers of PrP^Sc^, further corroborating the efficient inactivation of RML prions following a 12-h treatment with TiO_2_-based photocatalysis.

## 4. Discussion

Prion diseases pose a health risk due to their transmissibility and the ability of some prion strains to adapt to new hosts, with the additional risk of inter-species transmission. The disease-associated PrP^Sc^ protein has long been considered the main, if not the sole component, of the prion disease’s pathogenic agent and is capable of propagating itself through the template-induced conformational conversion of the PrP^C^ protein. Prions may contaminate surgical instruments or medical devices after use on infected patients or asymptomatic carriers. Moreover, their presence has been reported in transplants and human cadaver-derived pituitary hormones [7], as well as in biological fluids; importantly transplantations and blood transfusions have been shown to be a means of prion transmission, resulting in iatrogenic CJD cases [29]. An additional, previously underestimated, source of prion infectivity is represented by liquid medical waste, especially waste generated by pathology and biochemical laboratories, as well as slaughterhouses, where tissue and biological fluid processing takes place. Prion shedding in the environment by means of affected animal secretions, where it retains its infectivity for years, has also been reported [30]. The monitoring of potential prion sources and the development of efficient prion inactivation methods are urgently needed to reduce prion-related health risks.

Prions are extremely resilient to conventional inactivation methods, and despite extensive research, efficient prion inactivation methods are still needed. Previous studies from the past decades focused on the topic of prion inactivation [15]. So, different chemical compounds are reported to efficiently reduce prion infectivity by >3log10 within 1 h of treatment, such as chlorine (>1000 ppm), guanidine thiocyanate (>3 M), concentrated hydrogen peroxide (59%), a combination of hydrogen peroxide (100 mM) and copper (0.5 mM), sodium hydroxide (>1 N), alkaline detergent, and phenolic specific formulations. Additionally, autoclaving at 134 °C for 18 min is also reported to achieve an infectivity reduction of >3log10. Infectivity from artificially inoculated stainless steel wires with hamster scrapie 263K prions has been reported to be 100% reduced within 1 h of treatment with sodium hypochlorite (20,000 ppm), sodium hydroxide (1 N), autoclaving, as described above, as well as alkaline detergent and phenolic specific formulations [10].

In the search for alternative prion inactivation methods, we have been investigating the potential of photocatalytic oxidation approaches [21,22,23], with positive results. Extending our previous research to include additional prion strains, we undertook a comprehensive study to determine the efficiency of homogeneous and heterogeneous photocatalytic oxidation on RML prion inactivation in aqueous suspensions, utilizing concentrations of the pathogen similar or higher than the ones expected to be present in liquid medical waste.

For the photocatalytic experiments, we selected the RML mouse prion strain since it is an extensively studied and stable prion strain [31]. Further, the initial brain homogenate applied in the experiments was 0.1% *w*/*v*, which corresponds to 1 mg of brain tissue per 1 mL of liquid and an infectivity titer of approximately 10^3.5^xLD_50_. This amount is representative of real-life conditions, taking into consideration that the liquid waste we aimed to simulate generally contains only a small fraction of infectious material [32]. Since it is possible that prion strain variation may influence the efficacy of treatment approaches [33], the results of this study apply to RML prion strain in mice and cannot be easily adapted to every prion strain. Yet, in a recent study [34], it was proposed that the efficiency of each inactivation method should be proven for each prion strain against which it is intended to be used. The limitation of the presented results concerning the adaptation to other prion strains stems mainly, according to our opinion, from the different aggregation states, which PrP^Sc^ of diverse species or strains may undergo [35,36]. Hence, the approachability of the very short-living ROS species (HO^•^ radicals have a lifetime of 10^−9^ s) to the differently aggregated PrP^Sc^ molecules [37] produced during photocatalytic treatment might be significantly influenced.

Comparing the results we obtained in the present study, with the efficacy of the chemical or thermal inactivation methods mentioned before, it is obvious that photocatalysis needs much longer than 1 h of treatment time (12 h) to achieve similar results. This feature is the main disadvantage of the proposed method. On the other hand, there are certain advantages compared to reported prion inactivation methods. Photocatalytic treatment is user- and environment -friendly since the usage of harsh chemicals, such as chlorine, guanidinium, and phenolic derivatives, and high energy consumption are avoided. In particular, TiO_2_ is a completely inert reagent, and both catalysts are inexpensive and can be reused for more photocatalytic cycles. 

Our data indicate that heterogeneous TiO_2_-based photocatalysis performs significantly better than homogeneous photocatalysis, at least in the setting of the present study. All nine mice of the “TiO_2_ 12 h” group, which were inoculated with infectious material that had been treated for 12 h, and seven out of nine mice of the “TiO_2_ 6 h” group survived until the termination of the bioassay, i.e., 415 dpi, while all positive control mice (“control TiO_2_” group) reached the terminal disease stage between 240 and 270 dpi. On the other hand, photo-Fenton treatment was less efficient in this context, with most of the mice from the “Fe^3+^ 6 h” group (8/9) developing the disease symptoms almost simultaneously with the positive control group (“Fe^3+^ control”) of mice. Prolongation of the treatment for an additional 6 h only marginally improved performance as six out of nine mice succumbed to the disease. 

The present study provides concrete evidence that RML scrapie prions may be inactivated by the application of photocatalytic methods. This may be especially interesting in handling prion-contaminated or potentially contaminated liquids. As shown in this study, prions from 100 mg L^−1^ brain equivalents of final-stage diseased mice could be efficiently inactivated after TiO_2_ photocatalytic treatment for 12 h. Future experiments are necessary to optimize reaction conditions, reduce reaction time, and demonstrate the scalability of the method. The development of an inactivation indicator is particularly important because it may simplify the monitoring of the desirable inactivation levels.

## Figures and Tables

**Table 1:** Summary of RT-QuIC results. The bioassay is considered to be positive when the mouse reaches the disease terminal stage, while the RT-QuIC assay is considered to be positive when the brain homogenate contains PrP^Sc^ that drives recPrP conversion and aggregation.

Group	Sample	Bioassay	RT-QuIC
FeCl_3_–Control	1	+	+
FeCl_3_–Control	2	+	+
FeCl_3_–Control	3	+	+
FeCl_3_–6 h	1	+	+
FeCl_3_–6 h	2	+	+
FeCl_3_–12 h	1	+	+
FeCl_3_–12 h	2	+	+
FeCl_3_–12 h	3	-	-
TiO_2_–Control	1	+	+
TiO_2_–Control	2	+	+
TiO_2_–Control	3	+	+
TiO_2_–6 h	1	-	-
TiO_2_–6 h	2	-	-
TiO_2_–6 h	3	-	-
TiO_2_–12 h	1	-	-
TiO_2_–12 h	2	-	-
TiO_2_–12 h	3	-	-

## Data Availability

The original contributions presented in the study are included in the article, further inquiries can be directed to the corresponding authors.

## Supplementary Materials

The following supporting information can be downloaded at: https://www.mdpi.com/article/10.3390/pathogens13050420/s1, Table S1: Clinical symptoms of the scrapie disease in mice and the score they are accounting for the calculation of the clinical score; Figure S1: Data of the clinical evaluation of the mice subjected to the bioassay. References [38,39] are cited in the Supplementary Materials.
