# Supplementary material for: A Comparison of RML Prion Inactivation Efficiency by Heterogeneous and Homogeneous Photocatalysis
Source: Pathogens. 2024 May 16;13(5):420. doi: 10.3390/pathogens13050420 (PMC11124347; doi:10.3390/pathogens13050420)
Supplement: Supplementary file 1 [file pathogens-13-00420-s001.zip › pathogens-2972347-supplementary.pdf]

Supplementary Material

Clinical evaluation

Two observers blinded to the inocula administered to the mice performed the clinical evaluation, assessing the symptoms listed on Table 1 [1,2]. Mice with a clinical score >1 were considered to be RML symptomatic.

Supplementary Table S1. Clinical symptoms of the scrapie disease in mice and the score they are accounting for the calculation of the clinical score.

Clinical Symptom		Score
Aberrant Walking (Duckwalking)	None	0
	Present	1
Motor Coordination (Walking on bars)	Normal	0
	Occasional False Steps	0.5
	Severe Difficulty	1
Strength	Normal	0
	Occasional Falling off the bars	0.5
	Inability of holding on to the bars with their hind legs	1
Self-grooming	Clean genitals	0
	Dirty genitals	1
Tremor	None	0
	Present	1
Return to upright position	Instant	0
	Slow	1
Hunched posture	None	0
	Present	1

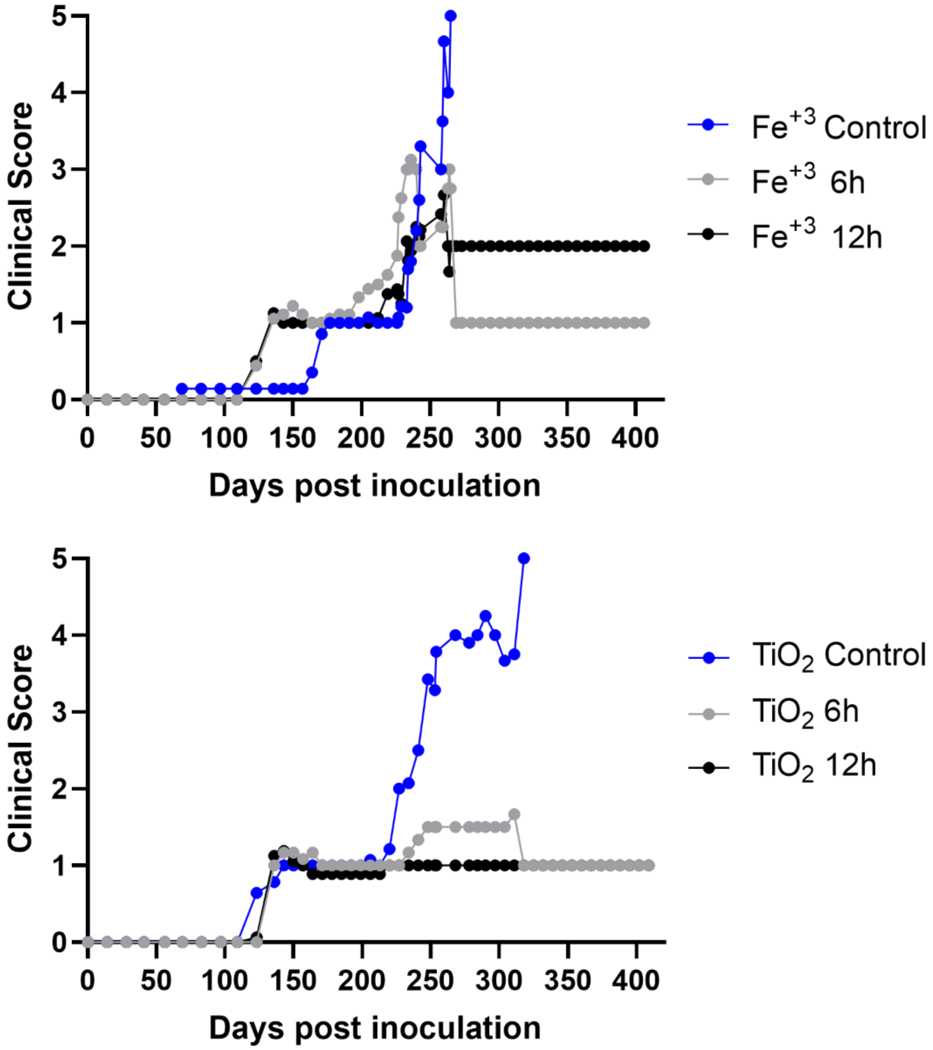

Supplementary Figure S1. Data of the clinical evaluation of the mice subjected to the bioassay. The average clinical score was plotted versus time for mice receiving material treated in the presence of the photo-Fenton reagent ($FeCl_3$, homogeneous photocatalysis, top panel) or TiO_2 (heterogeneous photocatalysis, bottom panel) for 6 or 12 h. Control mice received RML brain homogenate treated for 12 h in the absence of UV-A illumination. All mice presenting a clinical score <3 survived without the development of any other symptoms until the termination of the experiment.

References

1. Chiesa, R.; Piccardo, P.; Ghetti, B.; Harris, D.A. Neurological Illness in Transgenic Mice Expressing a Prion Protein with an Insertional Mutation. *Neuron* **1998**, *21*, doi:10.1016/S0896-6273(00)80653-4.
2. Moser, V.C. Functional Assays for Neurotoxicity Testing. *Toxicol Pathol* **2011**, *39*, doi:10.1177/0192623310385255.
